# Acute and sub-acute toxicity assessment of methanolic stem bark extract of *Khaya anthotheca* (Meliaceae) in Wistar rats

**DOI:** 10.1186/s41182-025-00721-9

**Published:** 2025-08-29

**Authors:** Betty Akwongo, Esezah K. Kakudidi, Anthony M. Nsubuga, Morgan Andama, Mary Namaganda, Patience Tugume, Savina Asiimwe, Godwin Anywar, Esther Katuura

**Affiliations:** 1 Department of Plant Science, Microbiology and Biotechnology, School of Biosciences, College of Natural Sciences, Makerere University, P. O. Box 7062, Kampala, Uganda; 2 Department of Biology, Faculty of Science, Muni University, P.O. Box 725, Arua, Uganda; 3 Ethnopharmacology and Zoopharmacognosy, Bernhard Nocht Institute for Tropical Medicine, Hamburg, Germany

**Keywords:** Acute toxicity, Sub-acute toxicity, Toxicity, Medicinal plants, *Khaya anthotheca*, Safety

## Abstract

**Background:**

*Khaya anthotheca* (Meliaceae) is a medicinal plant with a wide range of therapeutic properties attributable mainly to the diverse limonoids it contains. Different parts of the plant are used in traditional health care for treatment of various diseases including candidiasis. However, inadequate information on its safety prompted this particular study.

**Methods:**

Acute toxicity was assessed according to OECD guidelines 425 in female rats administered with single oral doses of 2000 and 5000 mg/kg body weight (b.wt), and monitored for 14 days for any sign of toxicity and mortality. Sub-acute toxicity was evaluated in both male and female rats following OECD guideline 407, and were administered with extract doses of 500, 250 and 125 mg/kg b.wt repeatedly for 28 days. Body weights were measured weekly, while food and water intake were measured daily. Blood for biochemistry, hematology, and organs for histopathology were collected at the end of the experimental period. Data were analyzed using one-way ANOVA followed by Turkey’s post hoc tests, and repeated measures ANOVA.

**Results:**

Acute toxicity showed no mortality, with half-lethal dose (LD_50_) being greater than 5000 mg/kg b.wt. For sub-acute toxicity, both male and female rats presented significant increase in food and water consumption, increased body weight with increasing time and extract doses (*p* < 0.05). The 500 mg/kg dosed female rats showed significant increase in stomach weights and alanine aminotransferase (ALT), while renal function marker of chloride ions (Cl^−^) decreased. Male rats showed dose-dependent significant rise in albumin (ALB) (*p* = 0.024). For both male and female rats, prolonged use of high extract doses of 250 and 500 mg/kg b.wt for 28 days were toxic to the stomach and liver.

**Conclusions:**

The methanolic stem bark extract of *K. anthotheca* is practically non-toxic at acute dose of 5000 mg/kg b.wt, and safe for clinical use at low sub-acute doses of 125 mg/kg b.wt. However, long-term administration of high extract doses above 125 mg/kg was toxic to mainly the liver and stomach. Thus, long-term administration of high dosage of methanol stem bark extract of *K. anthotheca*, and phytomedicine development should be done with cautions of potential side effects.

**Supplementary Information:**

The online version contains supplementary material available at 10.1186/s41182-025-00721-9.

## Background

*Khaya anthotheca* (Welw.) C.DC. is a large evergreen tree species in the family of Meliaceae. It is commonly known as the East African mahogany, and is native to tropical Africa [1]. Plants in genus *Khaya* including *K. anthotheca* exhibit a wide range of health promoting properties mainly due to presence of limonoids, which are highly oxygenated triterpenoids located in different parts of the plants with diverse antimicrobial activities [2]. The abundance of limonoids in genus Khaya has made these plants to be highly used as traditional herbal remedies, for example, Khaya species possess anti-inflammatory, antimalarial, anticancer, hepatoprotection, neuroprotection, antifeedant and antimicrobial properties [2]. Ethnobotanical survey by Akwongo et al. [3] documented *K. anthotheca* for treatment of candidiasis in Pader district in Northern Uganda. Verification of antifungal potential of the methanolic stem bark extract of *K. anthotheca* showed that, the extract possess broad spectrum antifungal activity against both fluconazole susceptible and resistant strains of selected *C. albicans*, *C. glabrata* and *C. tropicalis* [4], thus a promising plant template for novel antifungal drug development.

Despite the wide use of genus Khaya including *K. anthotheca* by different communities for treatment of several ailments including fungal infections, limited studies ascertained safety of these therapeutic plants. Medicinal plants are often considered to be safe because they are from natural sources. However this may not always be the case due to many reported cases of herbal toxicity [5, 6]. For example, volatile extract of *Khaya grandifoliola* presented high level of cytotoxicity in erythrocytes due to high concentration of sesquiterpenes [7]. Onu et al. [8] also reported organ-specific toxicity of *Khaya senegalensis*. Normally the rat model is recommended for chemical toxicity evaluation [9, 10] due to its close resemblance to humans’ genetic constitution and physiological processes [11]. Thus, the aim of this study was to determine the toxicity of *K. anthotheca* in Wister rats, an antifungal plant widely used in Northern Uganda for treatment of common candidiasis [3]. This was to predict safe doses of the extracts for human use, thus, promoting best practices in herbal medicine use to protect public safety, and to guide drug regulation authorities for further standardization and development into antifungal drugs. This is envisioned to curb rising cases of antifungal drug resistance to the few available drugs in the market. It will also provide information to justify conservation needs of this plant species for its sustainable utilization for provision of primary health care needs.

## Methods

### Plant materials

Fresh stem barks of *K. anthotheca* (Welw.) C.DC. plants were collected from Ogul village, Pajule subcounty in Pader district, Northern Uganda (03° 0′22″N, 33°1′43″E; altitude 1080.9 m). Plant sample collection was done in accordance to the IUCN policies that deals with research involving species at risk of extinction, just like *K. anthotheca* species [12]; minimum amount of fresh bark of *K. anthotheca* that was necessary for the accomplishment of the research were collected to reduce on the over utilization of the plant materials. Sample collection was done in the morning hours according to the community practice to preserve the plant’s therapeutic quality. Non-lethal sampling methods of vertical strip bark harvesting method with 2 collection frequencies on a plant stem was used, so as to minimize damage to the plants and facilitate bark recovery [13]. The plant voucher specimen was identified at Makerere University Herbarium; voucher number MHU51230, collection number BA013 [3].

### Preparation of methanol stem bark extract of *K. anthotheca*

Air dried stem barks of *K. anthotheca* were powdered and used to prepare methanol extracts according to Thirumalai et al. [14]. One hundred (100) grams of the powder was measured using Ohaus Triple beam balance (USA) and soaked in 1000 mL of 60–90% petroleum ether in a closed glass bottle for 72 h with regular shakings, after which the supernatant was decanted. Petroleum ether solvent was evaporated from the powdered plant residue by air drying for 12 h. Subsequently, the defatted plant powder was subjected to methanol extraction by soaking the sample in 1000 mL of methanol (Lichrosolv^R^ Germany) in a closed glass jar, and the same extraction procedure for petroleum ether was used to obtain supernatant which was filtered and concentrated under reduced pressure by rotary evaporation at appropriate temperature (35–40 °C). Extract yield of 62.49 (g) of dry extracts were obtained, and placed in stoppered sample vials and maintained at 4 °C, until use for toxicity tests.

### Experimental animals

All aspects of the animals ranging from selection, housing, grouping and acclimatization, feeding and welfare were handled according to OECD guidelines [15]. Fifty-five healthy young adult Wistar rats, aged 8 to 12 weeks of both sexes were used for this study. Female rats used were nulliparous and non-pregnant. To avoid disparity in results, the animals were all obtained from Pharmaceutical and Toxicology Research Laboratory, under the Research Center for Tropical Diseases and Vector Control in the College of Veterinary Medicine, Animal resources and Biosecurity (CoVAB), Makerere University. The animals were randomly selected, marked on their tails for individual identification using a permanent marker and assigned to their experimental groups in polypropylene cages according to their doses. The animals were kept in cages for 7 days prior to the start of dosing to allow for acclimatization to their surroundings. The cages contained sterile paddy husks as bedding, 12 h of light/dark cycle of artificial lighting, temperature of 22 °C (± 3 °C) °C and 50–60% relative humidity. All the animals had access to conventional laboratory diets (standard rat chow) and tap water ad libitum during the acclimatization period. Prior to the experiment, the animals were denied food overnight but not water, and weighed using a sensitive balance for calculation of accurate treatment dosages. All experiments were performed while adhering to the National guidelines for use of animals in research and teaching [16]. Before commencement of the study, the protocol was approved by School of Veterinary Medicine and Animal Resources (SVAR) of Makerere University, Institutional Animal Care and Use Committee (IACUC), reference number #SVAR_IACUC/100/2022. The rats’ endpoint was set at about 20% body weight loss [17]. The study results were reported using the ARRIVE guidelines [18].

### Experimental design

#### Acute toxicity tests

Acute toxicity was carried out using 5 female rats per dose categories according to the up and down procedure of OECD guidelines 425 [15]. The limit test was employed since available literature by Onu et al. [8] reported that, aqueous stem bark extract of genus *Khaya* was safe at limit dose of 5000 mg/kg b.wt. Thus, a starting limit dose of 2000 mg/kg b.wt (below the estimated LD_50_) followed by 5000 mg/kg b.wt were used. The rats were fasted overnight from food but not water prior to the commencement of dosing with the extracts. Extract doses were prepared shortly prior experiment using distilled water and administered via oral gavage to the rats starting with 2000 mg/kg b.wt group, followed by 5000 mg/kg b.wt group sequentially based on response of the first group of treated rats after 48 h. Rats in the control group were dosed with same amount of vehicle (distilled water). Food but not water was withheld for 3 to 4 h. The rats were individually observed for immediate signs of toxicity, such as, mortality, changes in physical appearance (skin and fur, eyes and mucous membrane), behavioral pattern (Heartbeat, salivation, sleep, lethargy), signs of illness and injury (diarrhea, comma, tremors, convulsions) at least once during the first 30 min. Then periodical observations were made during the first 24 h, with special attention given during the first 4 h. The rats were further observed daily for 14 days for any signs of delayed toxicity.

#### Sub-acute toxicity test

Sub-acute toxicity test was carried out in accordance with the OECD guideline 407 [10]. Forty rats comprising both males and females (nulliparous and non-pregnant) were randomly picked and divided into four groups, each contained ten rats in subgroups of 5 males and 5 females. Groups 1 to 3 were dosed daily with extracts of 500, 250 and 125 mg/kg of body weight using oral gavage for 28 days. Group 4 was the control and received the same amount of the highest volume of vehicle. The doses for sub-acute toxicity were determined from LD_50_ being ˃ 5000 mg/kg from acute toxicity experiment. Due to manifestation of some toxic signs during the acute toxicity study, one-fifth of nearest LD_50_ dose (5000 mg/kg) was calculated (1000 mg/kg) and then twofold serially diluted to descending doses of 500, 250 and 125 mg/kg body weights according to recommendation guideline of OECD 407. The serially double-diluted doses provided appropriate doses to establish the cumulative effects of repeated extracts administration to the experimental animals [19]. The rats were monitored for 28 days for signs of toxicity like changes in fur, skin, eyes, mucous membranes, occurrence of secretions and excretions, autonomic activity (e.g., lacrimation, piloerection, pupil size, unusual respiratory pattern), changes in gait, posture and response to handling as well as the presence of clonic or tonic movements, stereotypies (e.g., excessive grooming, repetitive circling) or bizarre behavior (e.g., self-mutilation, walking backwards).

For both acute and sub-acute toxicity studies, bodyweights of individual rats were measured and noted previously on first day prior dosing, and subsequently weekly up to the end of the 14th and 28th days, respectively. Water and food consumption were measured and recorded daily for entire study duration and time of death if any, were recorded. At the end of each study, all the rats were fasted overnight, and on the 15th and 29th days, respectively, the final weights of the rats were measured and then anesthetized by exposing them to halothane vapor in a closed chamber, then about 3 mL of blood was collected using 3-mL syringes from each animal’s heart by performing cardiac puncture. For each blood sample, i) half of the blood was for hematological test, and ii) the other half of the blood was for biochemical test with same identification/labels. The rats were finally killed by cervical dislocation, with great care being taken to minimize suffering of the animals [20]. Vital body organs were harvested and weighed for gross pathology and histopathological examinations.

#### Hematological assessment

About 1.5 mL of each collected blood was put in well labeled heparinized tubes containing anticoagulant, ethylene diamine tetra-acetic acid (EDTA). Samples were placed in cool box and transported to Uganda Cancer Institute (upper Mulago) at Mulago National Referral Hospital. Hematological analyses were carried out with an automated hematology analyser Sysmex XNL-550, SN 14275. Parameters analyzed included red blood cell count (RBC), lymphocyte count (LYMPH), white blood cell count (WBC), hemoglobin concentration (HGB), monocytes count (MONO), neutrophils (NEU), eosinophils (EO), hematocrit (HCT), basophils (BASO), mean corpuscular hemoglobin (MCH), mean corpuscular volume (MCV), mean corpuscular hemoglobin concentration (MCHC), platelet count (PLT), immunoglobulin (IG), red cell distribution width—coefficient of variation (RDW-CV), red cell distribution width—standard Jegnie deviation (RDW-SD), platelet distribution width (PDW), mean platelet volume (MPV), platelet-large cell ratio (P-LCR), procalcitonin (PCT) [17, 20].

#### Biochemical assessment

Approximately 1.5 mL of each collected blood samples for biochemical tests were placed gently in a correspondingly well labeled plain tubes (without anticoagulant) to prevent hemolysis of the blood cells. Samples were then placed in cool box and transported to Biochemistry laboratory at Uganda Cancer Institute (upper Mulago) at Mulago National Referral Hospital within 1 h. After 3 h, samples were centrifuged at 5000 r/min for 15 min. Micropipettes were used to extract out the plasma into fresher vials, which were kept in the refrigerator maintained at − 20 °C until the analysis. Automated biochemical assessment analyzer COBAS 6000 was used to determine levels of liver function markers viz; albumin (ALB), total proteins (TP), alanine aminotransferase (ALT), aspartate aminotransferase (AST), gamma-glutamyl transferase (GGT), bilirubin total (BILT), direct bilirubin (BILD), alkaline phosphate (ALP); and renal function markers viz; creatinine (CREJ), urea (UREA) and electrolytes excretion: sodium ions (Na^+^), potassium ions (K^+^), and chloride ions (Cl^−^) [21].

#### Histological assessment

The liver, kidney, heart and stomach [22] excised from each animal were fixed in 10% formalin for 24 h, and later processed from pathology department at CoVAB in Makerere University using an automatic tissue processor Leica TP 1020. Subsequently, the tissues were, respectively, cleared and impregnated with melted paraffin wax and xylene. The blocks were sectioned using rotary microtome to a thickness of 5 μm and then stained with hematoxylin and eosin (H and E), respectively, and mounted on the microscope slides for examination for any defects [21].

### Statistical analysis

Variations in body and organ weights, food and water consumptions, hematological and biochemical parameters for the different extract doses were ascertained using one-way analysis of variance (ANOVA), followed by Turkey’s post hoc test for multiple pair wise comparisons for the differences among the groups. Test results were statistically significant at *p* ≤ 0.05. The trend of the dependent variables of weights, food and water consumptions of the male and female rats at different doses, taken over time were established using repeated measures ANOVA. Multiple regression analyses were done for only parameters with significant variations in the dependent and independent variables, to find out overall relationships between a dependent/outcome variable and independent/ predictor variables with more than one parameter viz; doses, sex and treatment time in weeks. Histological assessments were descriptively presented.

## Results

### Acute toxicity of methanol stem bark extract of *K. anthotheca*

#### Clinical signs of acute toxicity of methanol stem bark extract of K. anthotheca

The single doses of 2000 and 5000 mg/kg did not cause any mortality. There were no major toxicity indicator clinical signs. Two out of five rats dosed with 5000 mg/kg displayed salivation after 10 min, which lasted for maximum of 32 min. Also 5 rats dosed with 2000 mg/kg felt asleep after 3–10 min, and it lasted for 9–80 min (Additional file 1).

#### Weekly body weights, food and water consumption of rats across extract doses and treatment time in weeks

Results in Table 1 indicate no significant variation in food and water consumption across the control and experimental groups treated with varying extract doses for the 2 weeks. Body weights for both extract treated and control groups generally increased with no significant differences between the weights of the 5000 mg/kg dosed rats and the control (*p* ˃ 0.05), except weights for 2000 mg/kg dosed rats that reduced significantly in week 2 compared to the control (*p* = 0.005). However, the body weight decrease was less than 10% original body weight. Repeated measures ANOVA showed that the weight of the rats increased with increase in time/ weeks (F_(2,36)_ = 21.83; *p* = 0.000). A multiple regression analysis resulted in a significant model (F_(2, 42)_ = 13.046, *p* = 0.000, *R*^2^ = 0.383) with treatment time duration in weeks contributing significantly most to the weights of the rats (*p* < 0.05), but extract doses did not (*p* ˃ 0.05) (Additional file 2).

**Table 1:** Body weights, food and water consumption of rats after single exposure to *K. anthotheca* extract

Parameters	Week	Control	Dosages for the experimental groups		*p*-value
			2000 mg/kg	5000 mg/kg	
Weight (g), *n* = 5	Week0	79.90 ± 1.54	88.14 ± 1.91	81.98 ± 3.29	0.074
	Week1	90.48 ± 1.79	89.50 ± 2.61	94.88 ± 2.51	0.260
	Week2	107.14 ± 1.23^b^	87.10 ± 5.09^a^	100.80 ± 3.05^b^	0.005*
Food consumption (g), *n* = 5	Week1	12.52 ± 0.81	11.82 ± 1.42	14.23 ± 2.33	0.575
	Week2	14.83 ± 2.05	10.49 ± 1.77	12.46 ± 1.57	0.262
Water consumption (g), *n* = 5	Week1	10.10 ± 1.09	11.57 ± 1.40	10.94 ± 1.35	0.722
	Week2	12.64 ± 0.93	11.60 ± 1.59	10.87 ± 1.14	0.609

Values are expressed as mean ± SD (*n* = 5)

*Significantly affected parameter (*p* < 0.05), where superscripts of same letter codes in a row denote no significant difference, while different letters indicate significant differences among pairs of dosages, and dosages with the control, n = number of rats

#### ***Organ weights of rats across extract doses***

There were no significant differences in the weights of majority of selected internal organs of control and extract treated rat groups, except for the weight of the kidneys in 2000 mg/kg dosed rats that was statistically lower than that of the control (*p* = 0.014) (Table 2).

**Table 2:** Acute toxic effects of *K. anthotheca* extracts on the organ weights of rats across doses

Organ weight (g), *n* = 5	Control	Dosages for the experimental groups		*p* value
		2000 mg/kg	5000 mg/kg	
1. Liver	4.84 ± 0.17	4.18 ± 0.36	4.84 ± 0.23	0.168
2. Kidney	0.58 ± 0.02^a^	0.44 ± 0.02^b^	0.52 ± 0.04^ab^	0.014*
3. Heart	0.42 ± 0.04	0.34 ± 0.02	0.44 ± 0.02	0.082
4. Stomach	1.96 ± 0.16	1.88 ± 0.12	1.80 ± 0.08	0.668

Values are expressed as mean ± SD (*n* = 5)

*Significantly affected parameter (*p* < 0.05), where superscripts of same letter codes in a row denote no significant difference, while different letters indicate significant differences among pairs of dosages, and dosages with the control, n = number of rats

#### Biochemical assessment: liver function markers across extract doses

There were no significant differences in the levels of ALB, TP, AST, BILT, BILD and ALP across all the extract treated and control groups*,* except GGT that significantly increased and ALT that significantly decreased in the 5000 mg/kg dosed rats compared to the control group. ALT also significantly increased in the 2000 mg/kg dosed rats compared to the control (Table 3).

**Table 3:** Acute toxic effects of methanol extract of *K. anthotheca* on levels of liver function markers

Biochemical parameters, *n* = 5	Control	Dosages for the experimental groups		*p* value
		2000 mg/kg	5000 mg/kg	
1. ALB (g/L)	42.58 ± 0.87	41.28 ± 0.79	42.92 ± 1.34	0.511
2. TP (g/L)	65.56 ± 1.10	64.86 ± 0.75	65.18 ± 2.20	0.946
3. ALT (U/L)	112.92 ± 11.29^a^	160.22 ± 7.11^b^	80.28 ± 4.16^c^	0.000*
4. AST (U/L)	336.62 ± 74.72	278.06 ± 84.10	226.58 ± 27.62	0.526
5. GGT (U/L)	−6.00 ± 0.32^a^	−6.00 ± 1.61^a^	−1.20 ± 1.32^b^	0.024*
6. BILT (umol/L)	1.74 ± 0.41	2.86 ± 0.71	1.30 ± 0.11	0.097
7. BILD (umol/L)	−0.54 ± 0.40	0.12 ± 0.67	0.20 ± 0.15	0.483
8. ALP (U/L)	267.60 ± 53.76	182.80 ± 58.74	236.20 ± 19.12	0.463

Values are expressed as mean ± SD (*n* = 5)

*ALB* albumin, *TP* total protein, *ALT* alanine aminotransferase, *AST* aspartate aminotransferase, *GGT* gamma-glutamyl transferase, *BILT* bilirubin total, *BILD* direct bilirubin, *ALP* alkaline phosphate

*Significantly affected parameter (*p* < 0.05), where superscripts of same letter codes in a row denote no significant difference, while different letters indicate significant differences among pairs of dosages, and dosages with the control, n = number of rats

#### Biochemical assessment: renal function markers across extract doses

All the renal function markers in the rats treated groups were within the normal ranges of the control groups (Table 4).

**Table 4:** Acute toxic effects of methanol extract of *K. anthotheca* on levels of renal function markers

Biochemical parameters, *n* = 5	Control	Dosages for the experimental groups		*p* value
		2000 mg/kg	5000 mg/kg	
1. CREJ (umol/L)	40.60 ± 4.39	35.20 ± 2.50	34.40 ± 3.98	0.462
2. UREA (mmol/L)	4.46 ± 0.66	5.66 ± 0.83	3.94 ± 0.18	0.177
3. Na^+^ (mmol/L)	143.80 ± 0.80	142.20 ± 1.20	141.20 ± 0.86	0.202
4. K^+^ (mmol/L)	6.75 ± 0.75	5.65 ± 0.53	5.59 ± 0.63	0.389
5. Cl^−^ (mmol/L)	100.52 ± 0.86	99.68 ± 0.46	98.24 ± 1.05	0.185

n = number of rats

*CREJ* creatinine, *UREA* urea, *Na*^*+*^ sodium ions, *K*^*+*^ potassium ions, *Cl*^*−*^ chloride ions

#### Hematological assessment across extract doses

Single oral doses of 2000 and 5000 mg/kg of methanol stem bark extract of *K. anthotheca* did not significantly alter the levels of most of the hematological parameters. Compared to the control group, only RDW-SD significantly lowered in the treated group of 2000 mg/kg (Table 5).

**Table 5:** Acute toxic effects of methanol bark extract of *K. anthotheca* on levels of hematological parameters

Hematological parameters, *n* = 5	Control	Dosages for the experimental groups		*p *value
		2000 mg/kg	5000 mg/kg	
1. WBC (10^3^/uL)	7.13 ± 2.16	7.47 ± 1.88	5.29 ± 1.27	0.667
2. NEUT (10^3^/uL)	1.01 ± 0.42	1.53 ± 0.44	0.74 ± 0.24	0.358
3. LYMPH (10^3^/uL)	5.12 ± 1.51	5.07 ± 1.51	3.92 ± 1.00	0.784
4. MONO (10^3^/uL)	0.68 ± 0.32	0.41 ± 0.17	0.36 ± 0.17	0.596
5. EO (10^3^/uL)	0.11 ± 0.03	0.10 ± 0.04	0.12 ± 0.05	0.913
6. BASO (10^3^/uL)	0.20 ± 0.05	0.36 ± 0.10	0.15 ± 0.03	0.110
7. IG (10^3^/uL)	0.03 ± 0.01	0.04 ± 0.01	0.01 ± 0.01	0.128
8. RBC (10^6^/uL)	8.16 ± 0.21	7.94 ± 0.80	8.52 ± 0.14	0.707
9. HGB (g/dL)	13.72 ± 0.37	13.42 ± 0.91	14.10 ± 0.27	0.723
10. HCT (%)	49.92 ± 1.24	46.42 ± 4.84	50.58 ± 1.38	0.586
11. MCV (fL)	61.20 ± 0.79	58.32 ± 0.58	57.36 ± 2.12	0.154
12. MCH (pg)	16.80 ± 0.18	17.24 ± 0.87	16.54 ± 0.21	0.647
13. MCHC (g/dL)	27.48 ± 0.25	29.58 ± 1.66	27.90 ± 0.36	0.319
14. RDW-SD (fL)	29.04 ± 0.44^a^	26.30 ± 0.70^b^	29.94 ± 0.51^a^	0.002*
15. RDW-CV (%)	15.68 ± 0.48^ab^	15.40 ± 0.52^b^	17.18 ± 0.35^a^	0.035*
16. PLT (10^3^/uL)	538.40 ± 104.57	430.80 ± 97.43	545.00 ± 94.37	0.666
17. PDW (fL)	8.52 ± 0.32	7.72 ± 0.17	7.93 ± 0.13	0.059
18. MPV (fL)	7.72 ± 0.12	7.40 ± 0.13	7.55 ± 0.07	0.181
19. P-LCR (%)	9.40 ± 0.88	7.32 ± 0.78	8.60 ± 0.68	0.208
20. PCT (%)	0.41 ± 0.08	0.32 ± 0.07	0.48 ± 0.02	0.215

Values are expressed as mean ± SD (*n* = 5)

*Significantly affected parameter (*p* < 0.05), where superscripts of same letter codes in a row denote no significant difference, while different letters indicate significant differences among pairs of dosages, and dosages with the control, n = number of rats; WBC—white blood cell count, NEU—neutrophils, LYMPH—lymphocyte count, MONO—monocytes count, EO—eosinophils, BASO—basophils, IG—immunoglobulin, RBC—red blood cell count, HGB—hemoglobin concentration, HCT—hematocrit, MCH—mean corpuscular hemoglobin, MCV—mean corpuscular volume, MCHC—mean corpuscular hemoglobin concentration, RDW-CV—red cell distribution width—coefficient of variation, RDW-SD—red cell distribution width—standard Jegnie deviation, PLT—platelet count, PDW—platelet distribution width, MPV—mean platelet volume, P-LCR—platelet-large cell ratio, PCT—procalcitonin

#### Gross pathology and histopathological analysis of the vital organs of the rats

**Fig. 1:**
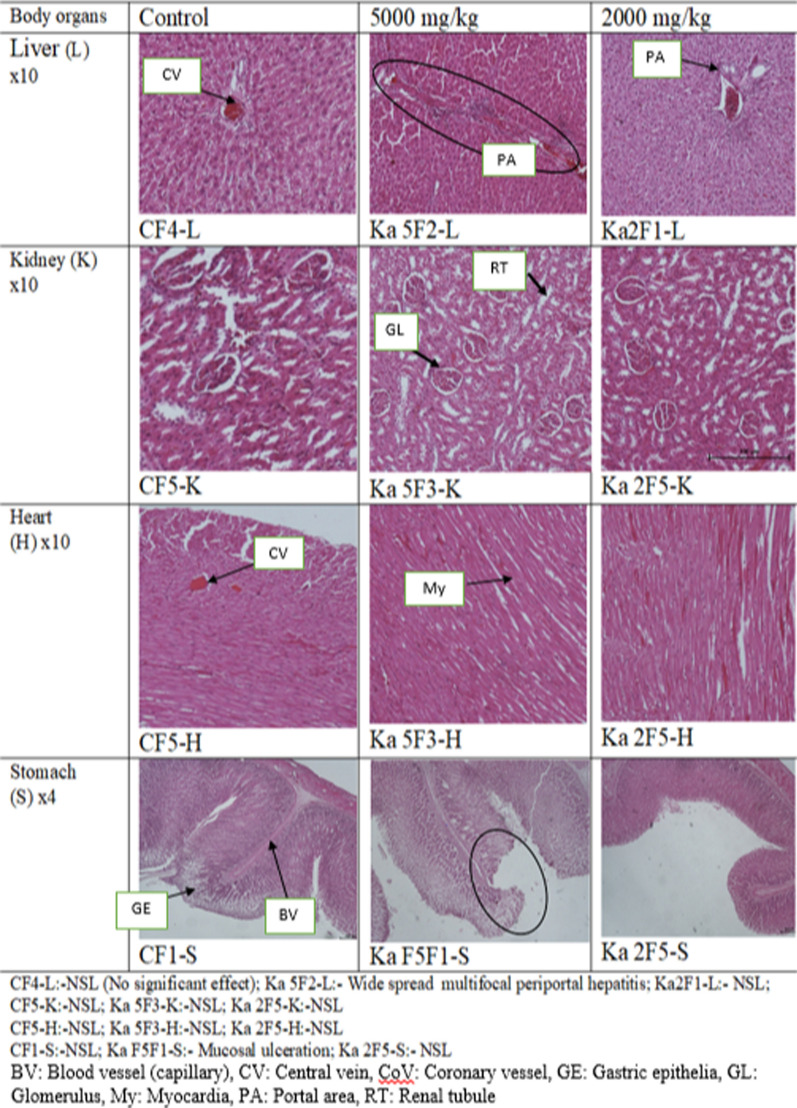
Photomicrographs of the liver, kidney, heart and stomach of the treated rats in acute toxicity

Gross pathology of the treated livers, hearts, kidneys and stomachs when compared to the control showed normal color, size, texture and shape, although two rats treated with 2000 mg/kg had a tumor in their liver lobes. Histopathological analysis showed no cellular difference between the treated and control groups for most of the organs, the tissues had normal architecture, except the liver that developed wide spread multifocal periportal hepatitis and the stomach with mucosal ulceration in rats treated with 5000 mg/kg (Fig. 1).

### Sub-acute toxicity of methanol stem bark extract of *K. anthotheca*

#### Weekly body weights, food and water consumption of rats across the extract doses, time in weeks and sex of the rats

With increasing extract doses, the weekly body weights of both males and female rats generally increased throughout the 4 weeks of experiment (Table 6). Although comparison by sex showed that the male rats were significantly heavier than the female rats (*p* < 0.05, Additional File 4). Repeated measures ANOVA showed a general increase in weights with increasing time in weeks (F_(4,160)_ = 73.15; *p* = 0.000). A multiple regression analysis resulted in a significant model (F_(3,196)_ = 151.837, *p* = 0.000, *R*^2^ = 0.699), with treatment time in weeks contributing significantly most to the weights of the rats, followed by extract doses and lastly the sex of the rats (*p* < 0.05) (Additional file 3).

**Table 6:** Sub-acute toxicity of *K. anthotheca* extract on rats’ weekly body weights, food and water consumption

Parameter	Wk	Sex	Control	Dosages for the experimental groups			*p*-value
				125 mg/kg	250 mg/kg	500 mg/kg	
Weight (g), *n* = 5	0	M	71.30 ± 1.49^a^	78.90 ± 1.24^b^	84.58 ± 1.81^b^	91.60 ± 1.94^c^	0.000*
		F	67.86 ± 1.94^a^	74.30 ± 0.76^ab^	80.04 ± 1.09^b^	89.52 ± 3.10^c^	0.000*
	1	M	78.84 ± 3.98^a^	89.30 ± 1.35^bc^	98.20 ± 2.23^c^	105.94 ± 1.23^c^	0.000*
		F	83.74 ± 2.24^a^	86.90 ± 1.09^a^	89.80 ± 2.74^a^	102.58 ± 4.02^b^	0.001*
	2	M	83.48 ± 6.24^a^	98.26 ± 3.04^ab^	108.06 ± 4.02^b^	116.12 ± 2.68^b^	0.000*
		F	91.32 ± 3.53	93.26 ± 3.00	98.72 ± 7.25	105.78 ± 4.04	0.177
	3	M	92.34 ± 9.09^a^	105.66 ± 7.16^ab^	121.36 ± 6.40^b^	126.10 ± 3.65^b^	0.011*
		F	99.36 ± 4.71	104.24 ± 2.73	109.04 ± 7.11	112.66 ± 4.46	0.297
	4	M	104.80 ± 1.49^a^	110.10 ± 6.14^ab^	128.50 ± 7.66^ab^	131.86 ± 5.84^b^	0.009*
		F	103.06 ± 6.97	107.70 ± 3.19	115.08 ± 8.69	116.40 ± 5.70	0.439
Food consumption (g), *n* = 5	1	M	11.97 ± 0.74^a^	14.60 ± 0.95^ab^	14.53 ± 0.44^ab^	15.79 ± 0.80^b^	0.018*
		F	17.10 ± 1.18^a^	12.55 ± 0.68^b^	14.11 ± 0.64^ab^	15.76 ± 0.60^ab^	0.006*
	2	M	12.34 ± 0.69	13.69 ± 1.25	14.88 ± 0.95	14.89 ± 1.50	0.366
		F	14.40 ± 1.00	13.78 ± 1.13	13.60 ± 1.00	14.18 ± 1.17	0.949
	3	M	11.48 ± 0.20^a^	11.29 ± 0.51^a^	15.82 ± 0.64^b^	15.48 ± 0.66^b^	0.000*
		F	13.35 ± 0.51	12.78 ± 0.68	14.56 ± 0.47	14.46 ± 0.72	0.148
	4	M	12.82 ± 0.60^a^	11.68 ± 0.63^a^	15.30 ± 0.62^b^	15.79 ± 0.40^b^	0.000*
		F	13.52 ± 0.17^a^	15.12 ± 0.17^b^	13.01 ± 0.28^a^	14.98 ± 0.32^b^	0.000*
Water consumption (g), *n* = 5	1	M	12.08 ± 0.59^a^	14.24 ± 1.17^a^	18.64 ± 0.98^b^	14.94 ± 0.93^ab^	0.001*
		F	13.70 ± 0.56^a^	10.74 ± 0.45^b^	12.48 ± 0.70^ab^	14.20 ± 0.48^a^	0.002*
	2	M	12.60 ± 0.57^a^	12.60 ± 0.37^ab^	15.44 ± 0.50^abc^	16.60 ± 1.34^c^	0.004*
		F	12.08 ± 0.83	10.88 ± 0.32	11.88 ± 1.01	13.12 ± 0.30	0.191
	3	M	14.48 ± 0.39	14.00 ± 0.79	15.44 ± 0.28	15.76 ± 0.28	0.070
		F	13.84 ± 0.44^ab^	12.48 ± 0.61^b^	17.48 ± 0.57^c^	15.48 ± 0.89^ac^	0.000*
	4	M	13.20 ± 0.64	13.88 ± 0.65	14.68 ± 0.56	15.28 ± 0.39	0.068
		F	12.44 ± 0.26^a^	11.52 ± 0.53^a^	14.88 ± 0.41^b^	15.20 ± 0.71^b^	0.000*

Values are expressed as mean ± SD (*n* = 5)

*Significantly affected parameter (*p* < 0.05), where superscripts of same letter codes in a row denote no significant difference, while different letters indicate significant differences among pairs of dosages, and dosages with the control, n = number of rats; M-male; F-female; Wk-week

Food consumption generally increases with increasing extract doses. For males, rats treated with 250 and 500 mg/kg consumed significantly more food compared to the control group in a dose-dependent manner (*p* < 0.05). In females, most treated rats did not show significant difference in food consumption compared to controls except in the fourth week, where rats treated with 125 and 500 mg/kg consumed more food than the controls (Table 6). Food consumption generally did not increase with treatment time in weeks using repeated measures ANOVA (F_(3,192)_ = 1.88; *p* = 0.135). A multiple regression analysis resulted in a significant model (F_(3,220)_ = 13.931, *p* = 0.000, *R*^2^ = 0.16), with extract doses significantly contributing most to food consumption (*p* < 0.05), but sex and weeks did not significantly influence food consumption in rats (*p* ˃ 0.05) (Additional file 3).

Water consumption generally increases with increasing extract doses for both treated males and female rats. Compared to the control, significant water consumption were recorded in the first and fourth week in males and females rats treated with 500 mg/kg, respectively, (*p* < 0.05) (Table 6). Comparison by sex indicated that males consumed more water than females (*p* < 0.05, Additional File 4). Repeated measures ANOVA results showed that water consumption generally increased with increasing weeks (F_(3,192)_ = 8.15; *p* = 0.000). A multiple regression analysis resulted in a significant model (F_(3,220)_ = 31.58, *p* = 0.000, *R*^2^ = 0.30), with extract doses contributing significantly most to water consumption of the rats, followed by sex and lastly treatment time duration in weeks (*p* < 0.05) (Additional file 3).

#### Organ weights of rats across sex and extract doses

In comparison with the controls, most organ weights of treated males and females rats did not significantly vary across extract doses, except sex-dependent significant increase in male kidney and female stomach in the 500 mg/kg dosed rats (*p* < 0.05) (Table 7). Comparison by sex indicated higher weight for males’ heart while females had higher stomach weight (*p* < 0.05, Additional File 4). A multiple regression analysis to predict variations in kidney and stomach of the rats by sex and extract doses, resulted in a significant model for stomach weights (F_(2, 37)_ = 15.798, *p* = 0.000, *R*^2^ = 0.461), and for kidney weights (F_(2, 37)_ = 3.869, *p* = 0.03 and *R*^2^ = 0.173. Further examination of the predictors of stomach weights showed that, overall, sex of the rats (females) contributed significantly most to the increased stomach weights (*p* < 0.05), followed by highest extract doses (*p* < 0.05). For the kidney weights, highest extract doses of 500 mg/kg administered to the rats contributed significantly most to the increased kidney weights (*p* < 0.05) while sex of the rats did not (*p* > 0.05) (Additional file 3).

**Table 7:** Sub-acute toxicity of *K. anthotheca* extract on the organ weights of rats

Organ weight (g), *n* = 5	Sex	Control	Dosages for the experimental groups			*p*-value
			125 mg/kg	250 mg/kg	500 mg/kg	
1. Liver	Male	4.66 ± 0.38	5.08 ± 0.28	5.00 ± 0.53	5.50 ± 0.49	0.599
	Female	5.06 ± 0.49	4.38 ± 0.11	5.44 ± 0.61	5.64 ± 0.39	0.232
2. Kidney	Male	0.48 ± 0.02^a^	0.46 ± 0.02^a^	0.54 ± 0.02^ab^	0.60 ± 0.03^b^	0.006*
	Female	0.52 ± 0.02	0.46 ± 0.02	0.50 ± 0.05	0.54 ± 0.02	0.412
3. Heart	Male	0.48 ± 0.06	0.46 ± 0.04	0.56 ± 0.04	0.58 ± 0.04	0.200
	Female	0.44 ± 0.02	0.46 ± 0.02	0.40 ± 0.03	0.46 ± 0.06	0.654
4. Stomach	Male	3.28 ± 0.16	2.80 ± 0.23	2.84 ± 0.29	2.38 ± 0.22	0.092
	Female	2.88 ± 0.18^a^	3.50 ± 0.35^a^	4.74 ± 0.26^b^	5.78 ± 0.27^b^	0.000*

Values are expressed as mean ± SD (*n* = 5)

*Significantly affected parameter (*p* < 0.05), where superscripts of same letter codes in a row denote no significant difference, while different letters indicate significant differences among pairs of dosages, and dosages with the control, n = number of rats

#### Biochemical assessment: liver function markers across sex and extract doses

Across the extract doses, the methanol stem bark extract of *K. anthotheca* did not cause significant variations in the levels of most liver function markers, except ALT which was significantly high in 500 mg/kg females treated rats (*p* = 0.002), and ALB significantly increased in 250 and 500 mg/kg treated male rats (*p* = 0.024) (Table 8). A multiple regression analysis to predict variations in ALT and ALB levels with sex and extract doses, resulted in models that were not significantly fit to explain ALT and ALB variations across sex and extracts doses (*p >* 0.05) (Additional file 3). Comparison of liver function markers by sex indicated that GGT and ALP were significantly higher in males while BILT was significantly high in females, although the mean values of these parameters were within normal ranges compared to the control, and did not statistically vary across the extract doses (Additional File 4).

**Table 8:** Sub-acute toxicity of *K. anthotheca* extracts on the liver function markers of the rats

Biochemical parameters, *n* = 5	Sex	Control	Dosages for the experimental groups			*p-*value
			125 mg/kg	250 mg/kg	500 mg/kg	
1. ALB (g/L)	M	43.78 ± 0.73	43.06 ± 1.42	47.36 ± 1.14	46.18 ± 0.33	0.024*
	F	42.92 ± 2.53	43.70 ± 0.80	44.14 ± 1.54	44.74 ± 1.50	0.894
2. TP (g/L)	M	71.48 ± 2.35	74.56 ± 2.07	75.08 ± 1.91	73.88 ± 0.51	0.544
	F	69.94 ± 1.43	70.42 ± 2.25	71.54 ± 2.12	73.82 ± 1.15	0.453
3. ALT (U/L)	M	113.40 ± 6.80	85.60 ± 8.61	92.72 ± 5.46	102.52 ± 6.75	0.062
	F	92.94 ± 5.36^ab^	83.28 ± 6.32^b^	108.98 ± 3.94^ac^	118.04 ± 4.92^c^	0.001*
4. AST (U/L)	M	194.68 ± 17.52	243.78 ± 44.21	189.40 ± 29.98	205.84 ± 21.65	0.586
	F	202.98 ± 27.76	206.48 ± 32.24	290.42 ± 55.33	283.46 ± 18.63	0.200
5. GGT (U/L)	M	−5.00 ± 1.34	−4.20 ± 5.98	4.40 ± 1.40	−7.80 ± 1.56	0.849
	F	−8.80 ± 0.66	−9.80 ± 1.77	−10.80 ± 0.86	−7.40 ± 1.36	0.290
6. BILT (umol/L)	M	1.26 ± 0.25	1.00 ± 0.34	1.04 ± 0.09	1.44 ± 0.17	0.529
	F	1.42 ± 0.13	1.53 ± 0.20	1.70 ± 0.16	1.90 ± 0.33	0.440
7. BILD (umol/L)	M	1.02 ± 0.24	3.80 ± 2.53	0.66 ± 0.13	0.64 ± 0.15	0.272
	F	0.42 ± 0.07	4.08 ± 2.90	0.76 ± 0.27	0.54 ± 0.07	0.263
8. ALP(U/L)	M	316.80 ± 45.42	265.80 ± 69.05	279.40 ± 42.95	264.80 ± 31.46	0.864
	F	138.40 ± 22.48	151.20 ± 9.30	190.60 ± 25.24	140.20 ± 15.89	0.229

Values are expressed as mean ± SD (*n* = 5)

*Significantly affected parameter (*p* < 0.05), where superscripts of same letter codes in a row denote no significant difference, while different letters indicate significant differences among pairs of dosages, and dosages with the control; n = number of rats; M-male; F-female

ALB- albumin, TP- total protein, ALT- alanine aminotransferase, AST- ASPARTATE aminotransferase, GGT- gamma-glutamyl transferase, BILT- bilirubin total, BILD- direct bilirubin, ALP- alkaline phosphate

#### Biochemical assessment: renal function markers across sex of the rats and extract doses

Across the extract doses, the methanol stem bark extract of *K. anthotheca* did not cause significant variations in the levels of the renal function markers, except Cl^−^ ions which was significantly low in females treated with 500 mg/kg (*p* = 0.006) (Table 9). A multiple regression analysis resulted in a significant model (F_(2, 37)_ = 17.968), *p* = 0.000, *R*^2^ = 0.493), with sex of the rats (females) contributing significantly to decrease in Cl^−^ ions levels (*p* < 0.05), extract doses did not (*p* ˃ 0.05) (Additional file 3). Comparison of the renal function markers by sex indicated that CREJ, K^+^, Cl^−^ and ALP were significantly higher in males while UREA was significantly higher in females (*p* < 0.05, Additional File 4), although the mean values of these parameters were within normal ranges compared to the control, and did not statistically vary across the extract doses (Table 9).

**Table 9:** Sub-acute toxic effects of methanol bark extract of *K. anthotheca* on the renal function markers

Biochemical parameters (mean ± SE, *n* = 5)	Sex	Control	Dosages for the experimental groups			*p-*value
			125 mg/kg	250 mg/kg	500 mg/kg	
1. CREJ (umol/L)	M	50.00 ± 5.40	63.20 ± 9.07	47.00 ± 4.29	50.00 ± 3.39	0.257
	F	38.80 ± 2.75	51.00 ± 6.88	41.00 ± 2.05	43.40 ± 3.08	0.218
2. UREA (mmol/L)	M	6.46 ± 0.10	6.36 ± 0.24	5.98 ± 0.30	6.90 ± 0.25	0.091
	F	7.12 ± 0.56	6.50 ± 0.30	7.24 ± 0.50	7.16 ± 0.32	0.621
3. Na^+^ (mmol/L)	M	222.20 ± 33.16	204.00 ± 19.01	154.20 ± 4.24	202.80 ± 31.28	0.285
	F	112.00 ± 2.55	114.60 ± 1.44	111.80 ± 2.15	113.80 ± 1.07	0.672
4. K^+^ (mmol/L)	M	8.97 ± 1.02	11.46 ± 1.93	8.24 ± 1.85	9.23 ± 1.37	0.529
	F	43.03 ± 4.09	40.20 ± 1.71	44.93 ± 2.77	43.90 ± 2.19	0.678
5. Cl^−^ (mmol/L)	M	154.20 ± 23.89	148.72 ± 11.15	123.30 ± 12.22	138.06 ± 21.49	0.644
	F	91.28 ± 0.64^a^	89.50 ± 0.41^ab^	90.56 ± 0.85^a^	87.46 ± 0.60^b^	0.004*

Values are expressed as mean ± SD (*n* = 5)

*Significantly affected parameter (*p* < 0.05), where superscripts of same letter codes in a row denote no significant difference, while different letters indicate significant differences among pairs of dosages, and dosages with the control; n = number of rats, M-male; F-female; CREJ- creatinine, UREA- urea, Na^+^- sodium ions, K^+^- potassium ions, and Cl^−^- chloride ions

#### Hematological parameters of the rats across sex of the rats and extract doses

Methanol stem bark extract of *K. anthotheca* did not cause any significant variations in the levels of all hematological parameters (Table 10) across all the extract doses. Comparison of the hematological parameters by sex indicated that BASO, RBC, HGB, HCT and MCHC were significantly higher in males while MCV, MCH, RDW- SD, MPV were significantly high in females (*p* < 0.05, Additional File 4), although the mean values of these parameters were within normal ranges compared to the control, and did not statistically vary across the extract doses (Table 10).

**Table 10:** Sub-acute toxicity *K. anthotheca* extract on the hematological parameters of the rats

Hematological parameters, *n* = 5	Sex	Control	Dosages for the experimental groups			*p *value
			125 mg/kg	250 mg/kg	500 mg/kg	
1. WBC (10^3^/uL)	M	10.51 ± 2.13	8.54 ± 1.55	9.12 ± 1.40	9.61 ± 2.08	0.887
	F	9.06 ± 3.12	6.66 ± 0.86	5.48 ± 0.96	12.38 ± 2.71	0.157
2. NEUT (10^3^/uL)	M	1.25 ± 0.05	0.99 ± 0.50	1.30 ± 0.21	1.11 ± 0.15	0.869
	F	1.74 ± 0.91	1.24 ± 0.26	1.02 ± 0.48	1.60 ± 0.47	0.811
3. LYMPH (10^3^/uL)	M	8.09 ± 1.82	6.74 ± 0.92	6.91 ± 1.15	7.47 ± 2.07	0.926
	F	6.67 ± 2.09	5.04 ± 0.95	4.04 ± 0.56	10.00 ± 2.27	0.093
4. MONO (10^3^/uL)	M	0.87 ± 0.34	0.50 ± 0.22	0.53 ± 0.24	0.46 ± 0.14	0.628
	F	0.46 ± 0.25	0.26 ± 0.10	0.25 ± 0.12	0.50 ± 0.09	0.558
5. EO (10^3^/uL)	M	0.07 ± 0.01	0.09 ± 0.03	0.16 ± 0.03	0.15 ± 0.03	0.073
	F	0.16 ± 0.06	0.09 ± 0.01	0.10 ± 0.02	0.20 ± 0.03	0.121
6. BASO (10^3^/uL)	M	0.24 ± 0.03	0.23 ± 0.09	0.22 ± 0.01	0.41 ± 0.11	0.239
	F	0.03 ± 0.01	0.04 ± 0.02	0.06 ± 0.02	0.08 ± 0.02	0.358
7. IG (10^3^/uL)	M	0.018 ± 0.005	0.096 ± 0.063	0.038 ± 0.012	0.024 ± 0.007	0.343
	F	0.032 ± 0.011	0.016 ± 0.005	0.026 ± 0.005	0.026 ± 0.010	0.610
8. RBC (10^6^/uL)	M	9.23 ± 0.07	9.20 ± 0.28	9.71 ± 0.15	9.29 ± 0.25	0.294
	F	8.02 ± 0.18	8.30 ± 0.19	8.21 ± 0.08	8.49 ± 0.09	0.172
9. HGB (g/dL)	M	15.38 ± 0.08	15.22 ± 0.39	16.12 ± 0.26	15.64 ± 0.33	0.176
	F	13.80 ± 0.40	14.38 ± 0.20	14.16 ± 0.29	14.40 ± 0.16	0.416
10. HCT (%)	M	52.80 ± 0.43	52.22 ± 1.48	55.08 ± 0.79	53.88 ± 0.92	0.216
	F	47.56 ± 1.40	50.22 ± 1.12	49.34 ± 0.90	51.32 ± 0.68	0.121
11. MCV (fL)	M	57.22 ± 0.19	56.80 ± 0.80	56.74 ± 0.19	58.10 ± 1.38	0.624
	F	59.30 ± 0.69	60.52 ± 0.87	58.04 ± 2.40	60.46 ± 0.63	0.545
12. MCH (pg)	M	16.68 ± 0.06	16.58 ± 0.19	16.62 ± 0.15	16.82 ± 0.35	0.870
	F	17.20 ± 0.23	17.36 ± 0.19	17.26 ± 0.23	16.96 ± 0.15	0.562
13. MCHC (g/dL)	M	29.10 ± 0.14	29.18 ± 0.13	29.26 ± 0.28	29.02 ± 0.24	0.859
	F	29.00 ± 0.13	28.66 ± 0.30	28.72 ± 0.25	28.06 ± 0.23	0.075
14. RDW-SD (fL)	M	27.16 ± 0.12	29.16 ± 1.28	28.10 ± 0.54	28.04 ± 0.77	0.394
	F	28.68 ± 0.42	29.96 ± 0.46	30.52 ± 0.97	29.50 ± 0.25	0.200
15. RDW-CV (%)	M	17.48 ± 0.04	18.40 ± 0.39	18.36 ± 0.17	17.52 ± 0.41	0.063
	F	17.38 ± 0.18	17.32 ± 0.43	17.72 ± 0.49	17.98 ± 0.23	0.541
16. PLT (10^3^/uL)	M	566.60 ± 5.19	448.60 ± 153.25	658.80 ± 46.43	581.40 ± 38.21	0.228
	F	638.00 ± 14.48	586.20 ± 104.36	501.60 ± 96.78	726.60 ± 44.03	0.232
17. PDW (fL)	M	7.40 ± 0.16	7.80 ± 0.53	7.34 ± 0.14	7.18 ± 0.14	0.510
	F	7.04 ± 0.14	7.76 ± 0.38	7.88 ± 0.43	7.78 ± 0.20	0.245
18. MPV (fL)	M	7.22 ± 0.07	7.16 ± 0.25	7.04 ± 0.08	7.04 ± 0.10	0.759
	F	7.22 ± 0.10	7.50 ± 0.24	7.62 ± 0.29	7.54 ± 0.08	0.533
19. P-LCR (%)	M	7.28 ± 0.44	7.08 ± 1.57	5.86 ± 0.52	6.08 ± 0.61	0.619
	F	6.46 ± 0.64	8.78 ± 1.94	9.18 ± 2.13	8.10 ± 0.44	0.598
20. PCT (%)	M	0.41 ± 0.01	0.31 ± 0.08	0.47 ± 0.04	0.41 ± 0.03	0.123
	F	0.46 ± 0.01	0.43 ± 0.07	0.37 ± 0.07	0.55 ± 0.03	0.162

Values are expressed as mean ± SD (*n* = 5)

n = number of rats, M-male; F-female; WBC- white blood cell count,NEU- neutrophils, LYMPH- lymphocyte count, MONO- monocytes count, EO- eosinophils, BASO- basophils, IG- immunoglobulin, RBC- red blood cell count, HGB- hemoglobin concentration, HCT- hematocrit, MCH- mean corpuscular hemoglobin, MCV- mean corpuscular volume, MCHC- mean corpuscular hemoglobin concentration, RDW-CV- red cell distribution width—coefficient of variation, RDW-SD- red cell distribution width—standard Jegnie deviation, PLT- platelet count, PDW- platelet distribution width, MPV- mean platelet volume, P-LCR- platelet-large cell ratio, PCT- procalcitonin

#### Gross pathology and histopathology

**Fig. 2:**
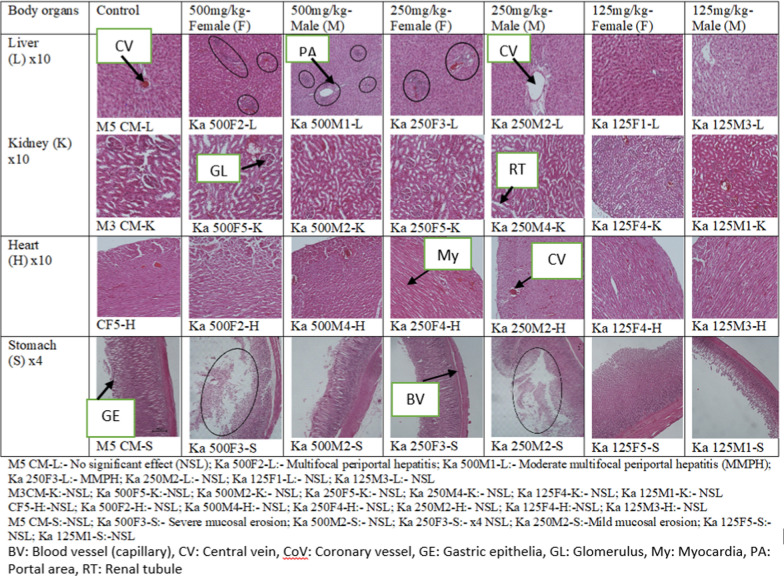
Photomicrographs of the liver, kidney, heart and stomach of treated rats in sub-acute toxicity

Gross pathology showed that, the methanol stem bark extract of *K. anthotheca* did not affect the gross pathology of the liver, heart, kidneys and stomach of the experimental rats. All organs had normal color, size, texture and shape when compared with those of the control groups (Fig. 2).

Histopathological analysis showed no cellular difference between the treated and controls groups for the heart and the kidneys, they had normal architecture. However, the extracts affected both the male and female stomachs and livers dose dependently. The 500 mg/kg treated female rats displayed more toxicology signs of mucosal erosion of the stomach and multifocal periportal hepatitis of the liver than the male counter parts. Safe dose of prolonged use of the extract to the vital organs of the animal mode (Wistar rats) is 125 mg/kg (Fig. 2).

## Discussion

Acute toxicity assessment of the methanol stem bark extract of *K. anthotheca* showed no severe morbidity or mortality of the rats single dosed with 2000 and 5000 mg/kg b.wt of *K. anthotheca* extract, indicating that the LD50 is > 5000 mg/kg b.wt. Thus, *K. anthotheca* is practically non-toxic to the rats up to the highest test limit dose of 5000 mg/kg as described in chemical toxicity classification by Hodge & Sterner [23]. The methanol stem bark extract of *K. anthotheca* can be placed in category 5 of acute toxicity according to Global Harmonized Classification System, since its LD_50_ is greater than 5000 mg/kg b.wt. This result agrees with the findings of Onu et al. [8] who reported LD50 of *K*. *senegalensis* to be > 5000 mg/kg b.wt.

Besides the relative safety, *K. anthotheca* demonstrating some mild toxic effects at 2000 mg/kg b.wt like induction of sleep behavior, and salivation by 5000 mg/kg b.wt dosed rats could be attributable to the phytochemicals of *K. anthotheca* acting on the serotonergic and gamma amino butyric acid (GABA) systems in the central nervous system. Thus promoting sedation and relaxation [24]. Herbal remedies toxicity can best be measured by evaluating hepatic and renal function marker toxicities. This is due to the high vulnerability of the liver to damage from drug toxic agents in the herbal drug concentrates and from metabolic function during detoxification [25]. Thus, evaluated liver function test was useful in determining the toxic effects of the phytomolecules to the animal model that would be extrapolated for safety if the herbal product is used in humans [26]. Similarly, the kidney is also highly vulnerable to toxins from herbs resulting from the high blood flow through the kidneys during urine concentration [25], as well as concentration of numerous substances in their tubular lumen [21].

Although the liver and kidneys are the primary organs affected by herbal toxicants; the heart is also a target organ for drug reactions and thus vulnerable to cardiac toxicity. Cardiac safety is used in pharmaceutical industry in attrition of drug candidates during pre-clinical phase of drug development [27]. The stomach is also at great risk of drug toxicity leading to mucosa ulceration that can result in serious complications of the gastro intestinal tract (GIT) [28]. Other measures of herbal toxicity included: blood parameters which have a higher predictive value for toxicity that can affect its diverse functions [26].

The large single dose administration of *K. anthotheca* did not affect any renal function markers implying that the extracts were safe, although there was reduction in the kidney weight. Changes in kidney weight is an important indicator of renal toxicity [29]. The kidney weights of the 2000 mg/kg dosed rats reducing by 24.1 signals possibility of developing hypertension due to increase in blood pressure [30]. Also, *K. anthotheca* did not affect majority of the liver function markers, hematology and histopathological parameters, except lowered RDW-SD which measures variation in the cell size of red blood cells, thus, important indicator of iron deficiency anemia [31]. However, RDW-SD reduction of ≤ 47 fl in 2000 mg/kg treated rats signifies non-severity of illness, therefore low risk of mortality [32]. There was alteration in the level of ALT enzyme, a highly sensitive key biomarker for monitoring structural integrity or damage of the liver tissues, thus, essential indicator for evaluating the safety of pharmaceutical products during drug development [33]. Increased ALT in 2000 mg/kg dosed rats indicates liver tissue damage [25], while decreased ALT in 5000 mg/kg dosed rats are attributed to increased frailty resulting in short life span, though the exact correlation between low ALT and early mortality is not so clear [34]. Perhaps, 5000 mg/kg dosed rats displaying organ-specific toxicity, i.e., widespread liver multifocal periportal hepatitis and stomach mucosal ulceration could account for early mortality of the individuals. This agrees with Singh et al. [35], who reported that drug-induced liver tissue damage account for about 10% human mortality. Additionally, acute mucosal ulceration is linked to significant mortality and morbidity [36]. There was change in the levels of GGT, a very sensitive enzyme used for the diagnosis of injuries in the liver [37]. Increased levels of GGT among the 5000 mg/kg dosed rats could possibly be due to damage in the liver or bile ducts leading to leaking of GGT into the bloodstream. This is in line with Ballotin et al. [38], who stated that herbal induced liver injuries are attributed to herbal metabolism. The acute toxicity of methanolic stem bark extract noted on Wistar rats is of great significance for standardization of its herbal products [39]. This will prevent herbal toxicity due to consumption of large single doses of the herbal products. Ethno botanical survey by Akwongo et al. [3] reported vomiting and inflammation of the Gastro Intestinal tract (like the mouth) as toxicity signs that manifests when anti-candida herbs including *Khaya anthotheca* are consumed in relatively large amount than the doses prescribed by the experienced herbalists.

Although *K. anthotheca* is practically non-toxic at the highest limit test dose of 5000 mg/kg, this may not guarantee its safety during sub-acute toxicity studies. This is due to reported cases of chemical compounds including phytocompounds being metabolized to toxic metabolites over time, which metabolites may bioaccumulate within the tissues of vital organs, thus resulting in organ-specific toxicity [39]. Thus, the need for conducting other tests like sub-acute toxicity to conclusively assess the toxicity of methanolic stem bark extract of *K. anthotheca* in the animal model used (Wistar rats). In sub-acute toxicity evaluation, daily dosing of rats with crude methanol bark extract of *K. anthotheca* up to 28 days did not result in morbidity or mortality, showing that the extracts may be relatively safe. This agreed with study carried out by Agbedahunsi et al. [40], who reported *Khaya ivorensis* to be relatively safe at lower doses.

For both males and females rats in varied extract doses, significant body weight increase by the experimental groups more than the control groups with increasing time/weeks due to more water but less food consumption, could be attributed to increasing accumulation of herbal toxins that induces stress [41]. The stress could have led to undereating [42], such that, the rats only consumed food required to meet their needs [43]. The excess calories consumed could have led to weight gain. Although comparison by sex indicate that males consumed more water and gained more weight, their mean values being similar to those of the control showed no sex-related toxicity. Nevertheless, the herbal toxicity shown by increased body weights may be insignificant since most of the body organs, biochemical, hematology and histopathology of the treated rats were within normal ranges with the control groups. Similarly, the herbal toxicity attributable to increased weights of male kidneys of rats dosed with 500 mg/kg could also be insignificant, since all the males’ biochemical and hematological parameters are within the normal ranges with the control.

However, long-term oral administration of methanol stem bark extract of *K. anthotheca* resulted in organ-specific toxicity mostly at high doses possibly due to accumulation of herbal toxic substances from the extracts. This agrees with a study findings on genus *Khaya* by Onu et al. [8], who found out that long-term administration of *K. senegalensis* resulted in organ-specific herbal toxicity. There was noted change in the level of Cl^−^ ions, an anion that regulates the volume of intracellular and extracellular fluids and maintains acid–base homeostasis [44]. Significantly decreased levels of Cl^−^ ions in 500 mg/kg dosed females signifies sex-dependent herbal toxicity of kidney functions of electrolyte balance, possibly due to females being more sensitive to toxic substances in methanolic extracts of *K. anthotheca*. García [45] noted that, females are more prone to chronic kidney diseases, although they are less likely to get end-stage kidney failure, like it is the case in males. Females are more prone to lupus which is an autoimmune disease that affects the immune system leading to damage of their tissues and organs like the kidneys, hence lupus nephritis greatly accounts for kidney infection-related deaths in women [46]. Relatedly, Onu et al. [8], also found out that, aqueous stem bark extract of *K. senegalensis* resulted in renal dysfunction that led to kidney electrolyte imbalance of sodium and potassium ions. However, methanol extract of *K. anthotheca* was safe to the kidneys of both male and female rats at low doses < 500 mg/kg since all toxicity parameters remained within normal ranges of the parameters for the control group of rats. Agbedahunsi et al. [40] also found out that high doses of ethanolic stem bark extract of *Khaya ivorensis* led to variations in kidney weights, which signified toxic effect on the kidney, but was safe at lower doses.

Significant increase in stomach weights for the 500 mg/kg dosed females rats, and its presentation with severe stomach mucosal erosion could be due to high sensitivity of female stomach walls that were in direct contact with phytomolecules in methanol stem bark extracts of *K. anthotheca* capable of causing ulceration of mucosal wall linings of the stomach. According to Lazic [47], significant organ weight change is an indication of organ damage by toxicity induced by chemical; which then proceeds to morphological distortion of the tissues [48], such as the tissue erosion of stomach mucosal lining.

Dose-dependent increased levels of ALT liver enzyme in highest 500 mg/kg dosed rats indicate liver tissue damage possibly by liver cytolysis. This was confirmed by histopathological slides of 500 mg/kg dosed rats presented with liver multifocal periportal hepatitis. These findings are in line with Abebe et al. [25], who found that liver tissue damage leads to the release of extra ALT liver enzyme to the blood circulation, thus making elevation in the levels of ALT an important biomarkers of liver tissue damage. Similarly, a study by Onu et al. [8] on liver toxicity of aqueous stem bark extract of *K. senegalensis* resulted in degeneration and necrosis of the liver cells, and degenerative function of the bile duct characterized by hyperplasia and fibrosis that led to lymphocytic infiltration of the hepatocyte. There was increased levels of ALT due to liver cytolysis, which were then released into rats’ circulation. Onu et al. [8] concluded that, since ALT enzymes are situated in the cytosol of the major parenchymal cells in the hepatocytes, their presence in blood circulation was a more hepatotoxic marker of liver damage caused by *K. senegalensis* than AST located in the mitochondria, cytoplasm and in the liver [8].

Similarly, there was noted change in the level of ALB, a protein that transports various substances throughout the body and prevents the fluids from leaking out of the blood vessels [49]. The dose-dependent increased levels of ALB in the 250 and 500 mg/kg dosed rats could be attributable to underlying health complications caused by toxins in the extracts. Albumin being a protein that is exclusively synthesized by the liver, makes it a marker of liver health and synthetic function. Thus, changes in the level of albumin signals degree of synthetic function of the liver [50]. According to Rozga, Piatek and Malkowski [51], albumin is a protein that carries out various roles including maintenance of plasma oncotic pressure by drawing water from the surrounding tissues into the blood vessels; albumin is also a transport protein to many substances like drugs, metabolites, hormones, enzymes, vitamins among others to tissue sites that require them, thus, changes in its levels is suggestive of health complications. Increased ALB is attributable to certain medications. Although more of other biochemical (GGT, ALP, CREJ, K^+^ and Cl^−^) and hematological parameters (BASO, RBC, HGB, HCT and MCHC) of the male rats were higher than those of the female rats, their mean values remaining within the normal ranges of those of the control groups is indicative of less serious sex-related toxicity for those specific parameters.

Despite the hepatic toxicity of Khaya species, this study demonstrated that *K. anthotheca* is safe to the liver at low doses. Overall, the methanol extract of *K. anthotheca* is safe at low doses of 125 mg/kg where all toxicity parameters remained at normal ranges with the control. This is in agreement with the study of Folarin et al. [52], in which, *Khaya senegalensis* was safe at only low doses.

### Limitations of the study

Use of Wistar rats from different parental stocks could have led to slight variation in tested parameters. However, control animals were used for each experimental group to counteract the limitations.

## Conclusion

The methanol stem bark extract of *K. anthotheca* extract was safe at the highest single dose of 5000 mg/kg of body weight. On repeated administration for 28 days, the extract was generally safe at low dose of 125 mg/kg of body weight. However, long-term treatment with higher extract doses of 250 and 500 mg/kg was toxic mainly to the liver and stomach. Thus, long-term administration of high dosage of methanol stem bark extract of *K. anthotheca* should be done with cautions of the likely side effects.

## Supplementary Information

Additional file 1

Additional file 2

Additional file 3

Additional file 4

## Data Availability

All data generated or analysed during this study are included in this published article [and its supplementary files].

## Abbreviations

ALB: Albumin
TP: Total proteins
ALT: Alanine aminotransferase
AST: Aspartate aminotransferase
GGT: Gamma-glutamyl transferase
BILT: Bilirubin total
BILD: Direct bilirubin
ALP: Alkaline phosphate
CREJ: Creatinine
UREA: Urea
Na^+^: Sodium ions
K^+^: Potassium ions
Cl^−^: Chloride ions
RBC: Red blood cell count
LYMPH: Lymphocyte count
WBC: White blood cell count
HGB: Hemoglobin concentration
MONO: Monocytes count
NEU: Neutrophils
EO: Eosinophils
HCT: Hematocrit
BASO: Basophils
MCH: Mean corpuscular hemoglobin
MCV: Mean corpuscular volume
MCHC: Mean corpuscular hemoglobin concentration
PLT: Platelet count
IG: Immunoglobulin
RDW-CV: Red cell distribution width—coefficient of variation
RDW-SD: Red cell distribution width—standard deviation
PDW: Platelet distribution width
MPV: Mean platelet volume
P-LCR: Platelet-large cell ratio
PCT: Procalcitonin
LD_50_: Lethal dose 50%
OECD: Organization for Economic Co-operation and Development
SE: Standard error
b. wt: Body weight
